# Attachment Patterns and OXTR Gene Methylation in Patients with Anorexia Nervosa and Their Mothers

**DOI:** 10.1192/j.eurpsy.2026.12029

**Published:** 2026-07-31

**Authors:** S. Ceran, B. Özel, A. Bağcaz, S. Akad Dinçer, Y. K. Terzi, M. Şenel, E. Akçay, A. Ö. Akman, Z. Yazıcıoğlu, O. M. Koçak

**Affiliations:** 1 Psychiatry, Başkent Univeristy; 2 Psychiatry, Hacettepe University; 3 Medical Genetics, Başkent Univeristy; 4 Psychiatry; 5 child and adolescent psychiatry; 6 Pediatrics, Ankara Bilkent City Hospital, Ankara; 7 Department of Psychiatry, Başkent University Department of Psychiatry, Istanbul, Türkiye

## Abstract

**Introduction:**

Anorexia nervosa (AN) is a severe eating disorder with a multifactorial etiology that remains incompletely understood, and has been linked to alterations in the oxytocin system. Methylation of the oxytocin receptor gene (OXTR) has been associated with social behavior and attachment patterns. Both theoretical and animal studies suggest that epigenetic patterns can be transmitted from parent to child. However, there is a notable lack of research on DNA methylation co-variation within parent–child dyads in AN.

**Objectives:**

This study aimed to examine methylation at six CpG sites (CpG29, CpG34, CpG35, CpG45, CpG46, and CpG47) located in the promoter and enhancer regions of the OXTR gene in women with active AN and their mothers, and to explore its associations with social behavior and attachment insecurity.

**Methods:**

The study was approved by the Başkent University Institutional Research Ethics Board, and all participants provided informed consent. AN diagnoses were based on DSM-5 criteria, and symptom severity was assessed using the Eating Attitudes Test-26 (EAT-26). Twenty-six mother–daughter dyads were recruited consecutively from a larger case-control study (daughters’ age: 15–25 years, mean ± SD: 17.7 ± 3.36; mothers’ age: 30–60 years, mean ± SD: 44.9 ± 7.2). The methylation status of OXTR at CpG sites 45, 46, 47, 29, 34, and 35 was assessed using Sanger sequencing following bisulfite conversion with region-specific methylation primers. Attachment dimensions were measured with the Turkish version of the Experiences in Close Relationships Scale (ECRS), and depressive and anxiety symptoms were evaluated using the Turkish versions of the Beck Depression Inventory (BDI) and Beck Anxiety Inventory (BAI).

**Results:**

Paired analyses revealed that daughters scored significantly higher than their mothers on both attachment dimensions (ECR-anxiety: 69.7 ± 27.8 vs. 58.8 ± 24.0, p = 0.043; ECR-avoidance: 71.2 ± 16.9 vs. 56.1 ± 19.6, p = 0.015). Using McNemar’s test, no significant differences in OXTR gene methylation frequencies were observed between mothers and daughters at CpG45–47 (100% vs. 100%), CpG29 (59.0% vs. 27.0%; p = 0.07), CpG34 (59.1% vs. 27.4%; p = 0.07), or CpG35 (59.1% vs. 27.3%; p = 0.07).

**Conclusions:**

Patients with anorexia nervosa exhibited higher attachment anxiety and avoidance scores compared with their mothers, despite high mean scores in both groups, suggesting that both attachment dimensions may be associated with the disorder. Methylation was detected in most of the six examined CpG sites, and no significant differences in OXTR gene methylation frequencies between mothers and daughters support potential transgenerational conservation of these epigenetic marks.

**Funding:**

This study was supported by the Health Institutes of Turkey (TUSEB), project number 35904, call code 2023-B-02.

**Disclosure of Interest:**

None Declared

